# A novel sensor design for accurate measurement of facial somatosensation in pre-term infants

**DOI:** 10.1371/journal.pone.0207145

**Published:** 2018-11-16

**Authors:** Alessandro Donadio, Kimberley Whitehead, Franck Gonzalez, Elisabeth Wilhelm, Domenico Formica, Judith Meek, Lorenzo Fabrizi, Etienne Burdet

**Affiliations:** 1 Department of Engineering, Università Campus Bio-Medico di Roma, Roma, Italy; 2 Department of Neuroscience, Physiology and Pharmacology, University College London, Gower Street, United Kingdom; 3 Department of Bioengineering, Imperial College of Science, Technology and Medicine, South Kensington Campus, London, United Kingdom; 4 Elizabeth Garrett Anderson Obstetric Wing, University College London Hospitals, United Kingdom; Chinese Academy of Medical Sciences and Peking Union Medical College, CHINA

## Abstract

Facial somatosensory feedback is critical for breastfeeding in the first days of life. However, its development has never been investigated in humans. Here we develop a new interface to measure facial somatosensation in newborn infants. The novel system allows to measure neuronal responses to touching the face of the subject by synchronously recording scalp electroencephalography (EEG) and the force applied by the experimenter. This is based on a dedicated force transducer that can be worn on the finger underneath a clinical nitrile glove and linked to a commercial EEG acquisition system. The calibrated device measures the pressure applied by the investigator when tapping the skin concurrently with the resulting brain response. With this system, we were able to demonstrate that taps of 192 mN (mean) reliably elicited facial somatosensory responses in 7 pre-term infants. These responses had a time course similar to those following limbs stimulation, but more lateral topographical distribution consistent with body representations in primary somatosensory areas. The method introduced can therefore be used to reliably measure facial somatosensory responses in vulnerable infants.

## Introduction

Facial somatosensation is critical for breastfeeding [1] and thus for appropriate weight gain in the first weeks of life [2]. In very pre-term infants, born up to three months early, breastfeeding can be suboptimal resulting in insufficient weight gain [3]. This is possibly because very pre-term birth can be associated with injury to afferent pathways, which may compromise the sensory feedback and cues normally provided by newborn feeding behaviours such as mouth gaping, licking and jaw jerking [4,5]. In line with this hypothesis, the use of pacifiers in pre-term infants improves feeding performance, potentially by somatosensory cortical reorganization compensating the deficit caused by the injury [6,7]. Currently, the development of facial somatosensation in humans is unknown, precluding a model of how this vital function establishes, and could be modified by pre-term birth, perinatal brain injury, or extra-uterine experience.

Somatosensory processing can be studied using electroencephalography (EEG). Peripheral stimulation elicits a sequence of deflections in the EEG called evoked potentials, that represent the activation of the cortical areas involved in the processing of the incoming afferent input [8,9]. Measuring the latency, amplitude and topographical distribution of this activity has a wide range of clinical and research applications related to the integrity and functioning of the somatosensory pathways [10]. Evoked potentials undergo relatively rapid changes over the equivalent of the last trimester of gestation as the brain matures [11,12]. Therefore, monitoring the development of these responses to touching the face could provide an insight into the maturation of neonatal facial somatosensation. However, existing stimulators are inadequate for facial stimulation in infants. Electrical and thermal stimulation are unethical for non-clinically required neonatal research protocols as they can extend into the nociceptive range [13,14]. Mechanical stimulation has been used as a suitable alternative for the stimulation of the limbs [15–18], but requires further caution and considerations to stimulate the delicate facial area and be accepted by the parents of these vulnerable subjects. Here we developed a new system to this end. This is based on a force transducer that an experimenter can wear on the finger underneath a clinical nitrile glove, linked to a commercial EEG acquisition system. The experimenter can then apply pressure on the skin of the subject while the sensing device provides a force measure time-locked with the neuronal activity. In this way the experimenter has full control of the magnitude and location of the force applied, providing a safe and versatile way to study facial somatosensory development in human pre-term infants.

## Materials and methods

### Circuit design considerations

To measure the cortical neuronal response of an infant to an experimenter’s touch, the system has to simultaneously record the force applied by the experimenter and the infant’s EEG. Moreover, it should fulfil the general requirements of any medical device, in particular be harmless and non-absorbent (can be wipe-cleaned with hospital detergent for infection control purposes).

An iterative design approach was used to develop the new device. We manufactured and tested an initial prototype (S1 Methods; S1 Fig), which led to the final device described here. The final design yields a sensorised finger consisting of i) a force transduction compartment and ii) a sensor-recording system interface.

### Force transduction

Force was transduced into a measurable resistance change with a standard piezoresistive force sensor (Flexiforce A301 from Tekscan). Key advantages of this sensor are i) its small size, leading to a compact measurement device that can be worn on the experimenter’s finger, ii) the simplicity of use, as it requires only a very simple conditioning circuit, and iii) the detectable force range that allows to record very light taps. A hemispherical rubber bumper (3M Bumpon) was affixed on the sensor surface with a pressure sensitive adhesive to evenly spread the load on the whole sensing area of the transducer.

### Sensor-recording system interface

EEG can be recorded with commercially available polygraphy systems, such as the NeuroscanTM SynAmps 2 used here. Such devices usually have auxiliary high voltage input lines to allow the concurrent recording of external signals. The resistance change provided by the piezoresistive force sensor was therefore converted into a voltage signal that could then be directly fed to the amplifiers of the polygraphy system.

The force sensor converts the force applied by the experimenter into variable resistance value *R*_*S*_ (*R*_*Smax*_ = 5MΩ), which, according to the typical performance curve of the A301 force sensor [19], is inversely related to the force:

$$R_{S}=\frac{k}{F}$$ (Eq 1)

where *k* is an unknown constant.

**Fig 1:**
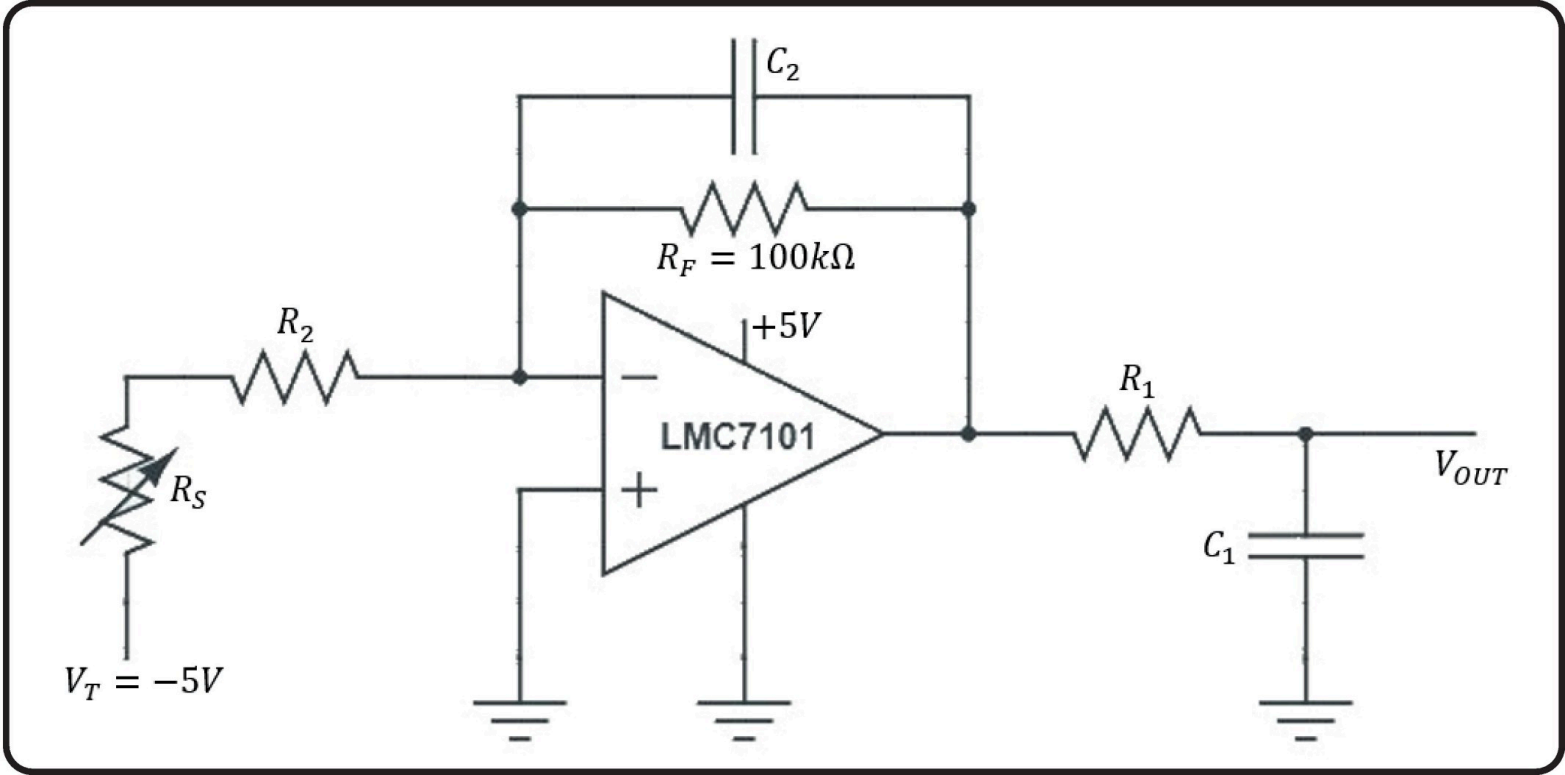
Recommended amplifier set-up configuration circuit for the A301 Flexiforce sensor (modified from[19]).

Changes in *R*_*s*_ are then converted into a voltage signal through an amplifier, which is set up as recommended in the A301 manufacturer’s datasheet (Fig 1). An RC low pass filtering circuit was also placed at the amplifier output.

In this configuration, the output voltage of the sensor-recording system interface in relation to *R*_*s*_ is:

VOUT(t,Rs)=VOUT(t=0)−VT(RF(jwC2RF+1)RS+R2)(1−e−1jwR1C1) (Eq 2)

which, considering a static or quasi-static load (i.e. ω = 0), can be simplified to:

$$V_{OUT}≅-V_{T}*\frac{R_{F}}{R_{S}+R_{2}}$$ (Eq 3)

where *V*_*T*_ and *R*_*F*_ are the voltage drive and the feedback resistor of the amplifier, respectively. The value of the feedback resistor *R*_*F*_ was chosen so that forces between 0–700 mN (i.e. corresponding to a gentle tap) spanned the full dynamic range of *V*_*OUT*_ (0 to +5V). Considering that in this force range, *R*_*s*_ ≫ *R*_2_, eq.3 can be approximated to:

$$V_{OUT}≅-V_{T}*\frac{R_{F}}{R_{S}}=-\frac{V_{T}R_{F}}{k}F$$ (Eq 4)

The +5V power supply for the amplifier (LMC7101 Texas Instruments) and the -5V voltage drive *V*_*T*_ were respectively obtained directly and through a charge pump inverter (TPS60403 Texas Instruments) from the auxiliary lines of the SynAmps 2 which provides a +5V power supply.

The whole system is powered at low-voltage (5V) and is very low power (below 1 mW) via the EEG system which is isolated from the main with an isolating transformer according to medical safety requirements.

### Development

**Fig 2:**
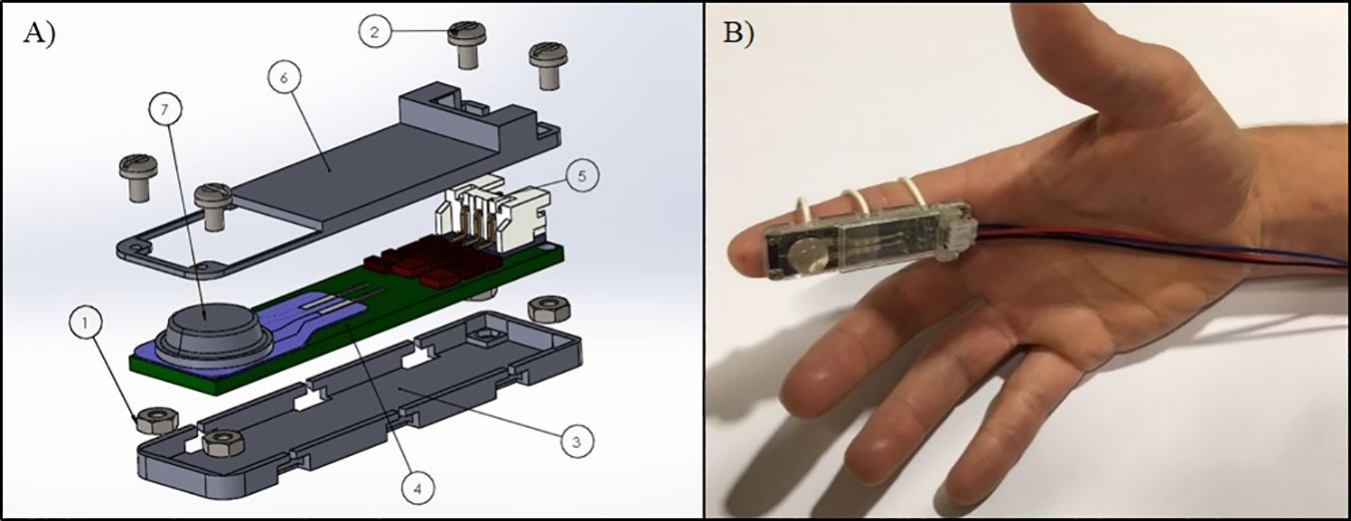
Sensorised finger device. A) 3D exploded CAD drawing of the casing for the device’s printed circuit board: 1) Hex M1x0.7mm Nuts; 2) M1 screws 0.4x3mm; 3) Lower case; 4) Printed circuit board; 5) JST Connector that links the device to the SynAmps2; 6) Upper case; 7) A301 Flexiforce sensor + 3M Bumpon. B) Sensorised finger as worn by a user.

A custom-made printed circuit board (PCB) was designed using Eagle CAD by Autodesk Inc. and manufactured by OSH Park (https://oshpark.com). The sensorised finger electronics consist of: two pin A301 Flexiforce sensor, charge pump inverter TPS60403 Texas Instruments, amplifier LMC7101 Texas Instruments, RC low pass noise filtering and three poles connector (+5V, Ground and *V*_*OUT*_) to link the device to an auxiliary line of the SynAmps 2 EEG system. The board is made on 2 layers with the sensor and all the components placed on the top layer. The pins of the A301 are directly soldered to the board providing a compact solution and a flat surface, which is necessary for the correct functioning of the transducer. The resulting board had compact dimensions (53.5x13x1.6 mm^3^) and could be encased in a small box of similar size (Fig 2A). The board and all its components were placed between an upper and a lower case, which are held together with four screws. The cases were printed using an “Objet30 Pro” 3D printer, which has a resolution of 0.1 mm required for the small dimensions of the box. The box has no sharp edges or corners. Three rubber bands placed in the lower case allow the user to wear the product under the finger, making the device suitable for different finger sizes (Fig 2B).

### Calibration

Calibration of the device was performed with a load frame (Instron 5866), by compression testing in 4 quasi-static trials (displacement controlled with a velocity of /) between 0 and 700 mN (corresponding to a gentle tap). The output voltage of the device was acquired at 1 kHz using a data acquisition board (National Instruments USB-6009). The linear force-voltage transfer function of eq.4 was then estimated using only the data from the loading part of the four trials, because tactile stimulation is performed by pressing the device on the skin starting from an unload condition.

### Clinical validation

Experiments were performed to test whether the device could be used to synchronise and measure the tapping of the skin with concurrent EEG recording allowing the analysis of evoked somatosensory responses. We first assessed whether we could reproduce existing results in the literature for tactile stimulation of the hand and foot, and then whether we could record responses to facial stimulation in pre-term infants.

#### Subjects

Seven infants were recruited for this study from the postnatal and neonatal wards at the Elizabeth Garrett Anderson wing of University College London Hospitals (four female) (S1–S7 Results). Each infant was born pre-term (mean gestational age at birth 34+6 weeks+days (range 31+0–36+3). Pre-term birth is defined as <37+0 weeks gestational age [20]. Each infant had a 5-minute APGAR score (index of clinical condition at birth) of ≥8/10. The mean corrected gestational age at study was 35+6 weeks+days (range 33+6–37+1), with mean postnatal age of 7 days (range 1–29). No neonates had congenital abnormalities, were acutely unwell, or receiving neuroactive medication at the time of study. Infants were neurologically normal both at the time of study and at the date of discharge based on review of medical notes and the discharge summary. Cranial ultrasound scans were reported as normal when subjects were referred for one (n = 6). All EEGs were assessed as normal for corrected gestational age by clinical scientist KW according to [21].

Ethical approval was obtained from the NHS Research Ethics Committee, and informed written parental consent was obtained prior to each study. Separate informed written parental consent was obtained to publish video recordings of two infants. The study conformed to the standards set by the Declaration of Helsinki guidelines.

#### EEG recording

Recording electrodes (disposable Ag/AgCl cup electrodes) were positioned according to the modified international 10/10 electrode placement system at F7, F8, F3, F4, Cz, CPz, C3, C4, CP3, CP4, T7, T8, P7, P8, TP9, TP10, O1 and O2 [22]. In a single infant the four posterior-temporal electrodes were omitted and estimated with spherical interpolation as implemented in EEGLAB. The reference electrode was placed at Fz [17,23–25] and the ground electrode was placed at FC1/2. Target impedance was <10 kΩ [26]. EEG was recorded with a direct current (DC)-coupled amplifier from DC-800Hz. Signals were digitized with a sampling rate of 2 kHz and a resolution of 24 bit.

#### Tactile stimulation

Gentle taps were delivered by clinical scientist KW to the lower cheek in all 7 infants, the palmar eminence of the hand in 6/7 infants, and the heel of the foot in 3/7 infants while they slept. Where possible both sides of the body were stimulated, although access to stimulation sites was limited by time constraints and infant positioning (handling was kept to a minimum) resulting in 13 datasets for cheek stimulation, 8 datasets for hand stimulation and 4 datasets for foot stimulation. We stimulated the cheeks rather than the lips because the lips are extremely sensitive in pre-term infants, with a small surface area, and their stimulation often evokes motor activity (e.g. reflex sucking/rooting) which can result in EEG artefact [5].

Taps were delivered while wearing the sensorised finger covered with a standard hospital-issue nitrile glove for infection control purposes (S1 Movie). The inter-stimulus interval was large, variable, and self-paced by the experimenter (5–15 s), as shorter intervals can attenuate somatosensory evoked potentials [27–29]. If the infant moved, the tap was delayed for several seconds to avoid potential modulation of the somatosensory response by motion [30] and to allow movement artefacts to resolve. The sequence in which the body areas were stimulated varied across subjects. We acquired a total of 25 stimulation trains of 8–45 stimuli each with a mean force of 192 mN (standard deviation 133 mN) (S2 Methods).

#### Analysis of somatosensory response

The occurrence of a tap was marked by thresholding the voltage generated by the force transducer (S1 Movie). Further data analysis was carried out using EEGLAB v.13 (Swartz Center for Computational Neuroscience) and custom-written MATLAB code. Line noise was removed with a 50 Hz notch filter (4th order Butterworth filter) and data were bandpass filtered at 1.5–40 Hz (2nd order Butterworth filter). Three datasets were de-noised using independent component analysis (an independent component representing ECG breakthrough was removed) [31]. Data were then epoched from -200 ms until +700 ms around the stimulus.

All EEG epochs were averaged across repetitions (i.e. each subject was characterised by a single average response per body area stimulated). Traces were aligned to the most prominent negative component to correct for intra-subject and inter-subject latency jitter with Woody filtering [32], alignment window: 130–180 ms (maximum allowed jitter -40 to +40 ms) (S2 Fig). Negative and positive components are denoted as N and P respectively. The presence of a somatosensory evoked potential in the grand average across subjects for each body area was determined as a significant deflection from baseline lasting >10 ms recorded at one or more of the contralateral mid-temporal, contralateral central, and midline central channels. Significant (p < .05) deflections were identified with a point-by-point t-test in which the mean at each time-point after stimulation (variance calculated for each channel and time point across repetitions) was compared against the mean of the baseline (variance calculated for each channel across time points and repetitions). To provide a visual representation of the topographical distribution of the somatosensory evoked potentials, we generated average traces and scalp maps for each body area (face, hand and foot).

## Results

### Calibration

**Fig 3:**
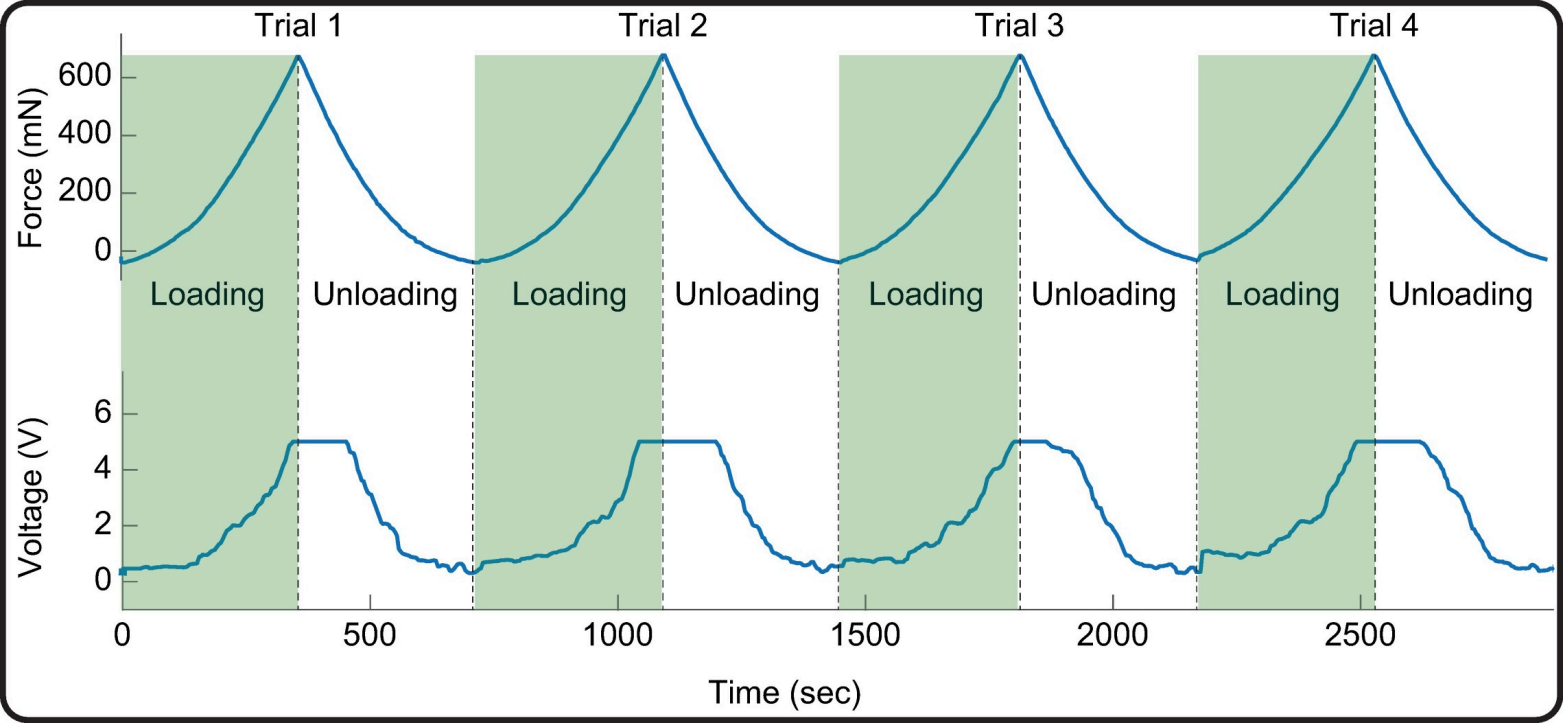
Sensor calibration. Upper panel: Compression test forces between 0–700 mN repeatedly applied in four quasi-static trials. Lower panel: Corresponding output voltage spanning the full dynamic range (0 to +5 V) and saturating for forces above approximately 650 mN. Green shading indicates the loading part of the four trials, from which the linear force-voltage transfer function of eq.4 was estimated.

The output voltage spanned the whole dynamic range (i.e. 0 to +5V) for forces between 0 and 700 mN saturating at approximately 650 mN (Fig 3).

**Fig 4:**
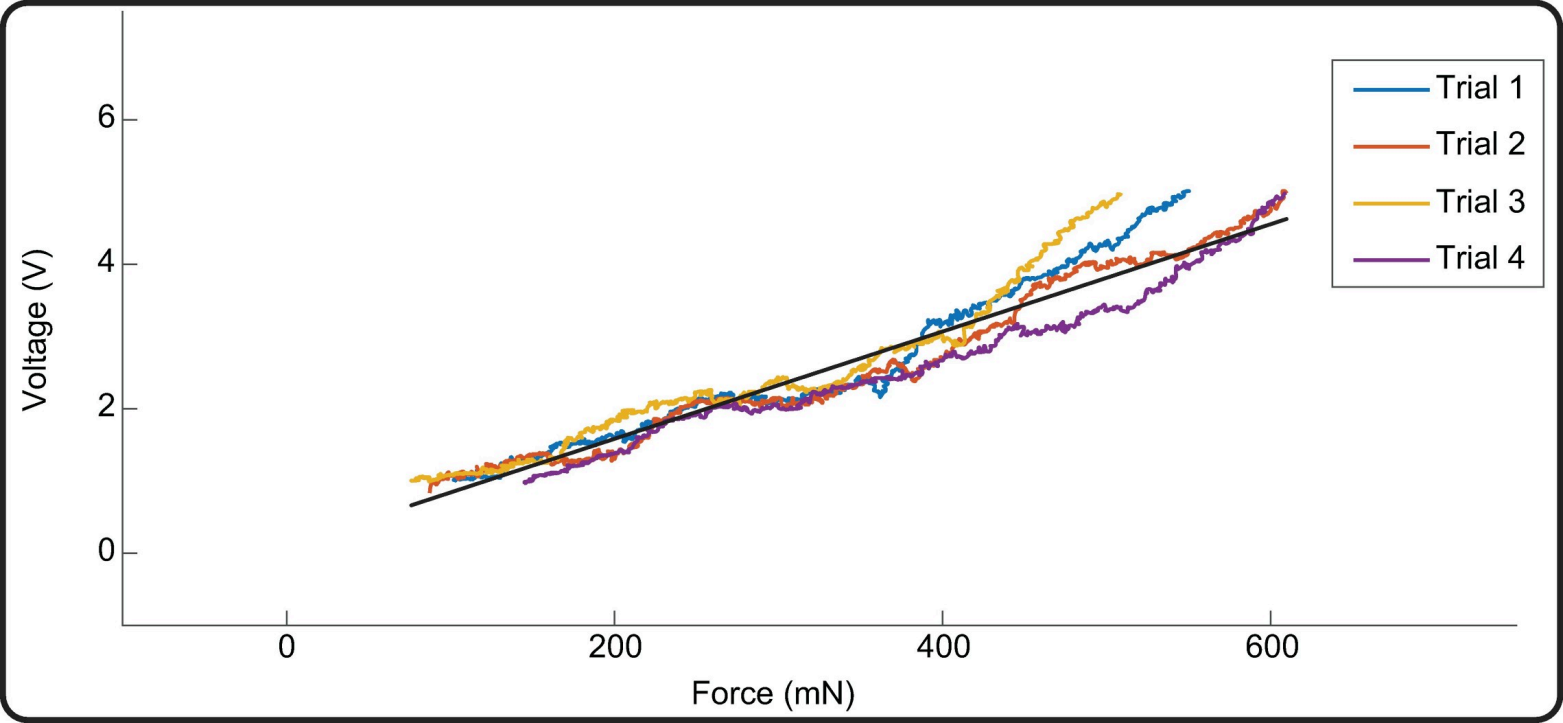
Force-voltage input-output transfer function. Voltage outputs at compression forces between 100 and 650 mN from the loading phases of four quasi-static repeated trials (coloured lines) and linear fit to the data (black line) representing the calibration curve of the device.

The loading part of the four compression trials was then used to estimate the force-voltage input-output transfer function of the device (Fig 4). This was a clear linear relationship (r^2^ = 0.94; p = 0.02) with equation:

$$V_{OUT}=m*F+q;\;\left\{ \begin{array}{c} m=6.957\;V/mN\\ q=0.278\;V \end{array}\right.$$ (Eq 5)

Forces below 100 mN were not considered, as this was the discrimination threshold of the sensor according to the A301 typical performance curve [19]. The precision of the device decreases with increasing loads (mean standard deviation of 11 mN for loads less than 450 mN and 36 mN for higher loads). Since the mean force applied in our clinical validation was 192 mN, experimental measurements are in the precise range.

### Clinical validation

Tactile stimulation using the wearable sensor was well tolerated by every infant. All seven infants slept through the whole protocol, and no reflexes were elicited (S1 Movie). Each parent was shown the device and found it acceptable, and the protocol did not interfere with nursing care. The mean force applied was lower than that obtained with an existing mechanical stimulator (modified tendon hammer) (192 vs. 267 mN) [33], confirming that allowing the experimenter to use her/his own hand to stimulate the skin allows delivery of more gentle pressure.

#### Recording of the applied force

**Fig 5:**
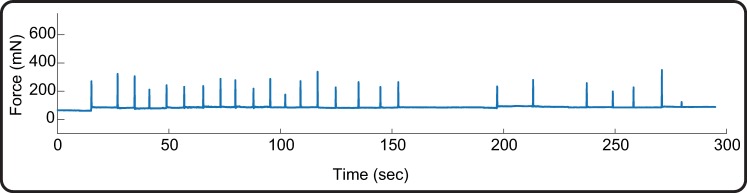
Example of force recording. Force recording from a train of 25 tactile stimuli delivered to the face. The tap occurrence is clear and can be easily detected. In this example, the baseline force has an offset of approximately 80 mN due to the glove worn by the experimenter pressing on the sensor.

The sensorised finger could record the force applied with each tap (Fig 5). The glove worn by the experimenter pressing on the sensor causes a variable baseline offset, however the force applied with each tap can be calculated as the absolute value of the peak force minus the offset. This is estimated as the average value of the force in the 0.5 s before the occurrence of that event.

Using the force recording we could mark the occurrence of each tap in all infants on the concurrent EEG (S1 Movie). We were then able to epoch the EEG according to the event timing, and average responses within subjects and across subjects.

#### Simultaneous Force-EEG recording allows to analyse cortical responses synchronised to tapping of the hand and foot

**Fig 6:**
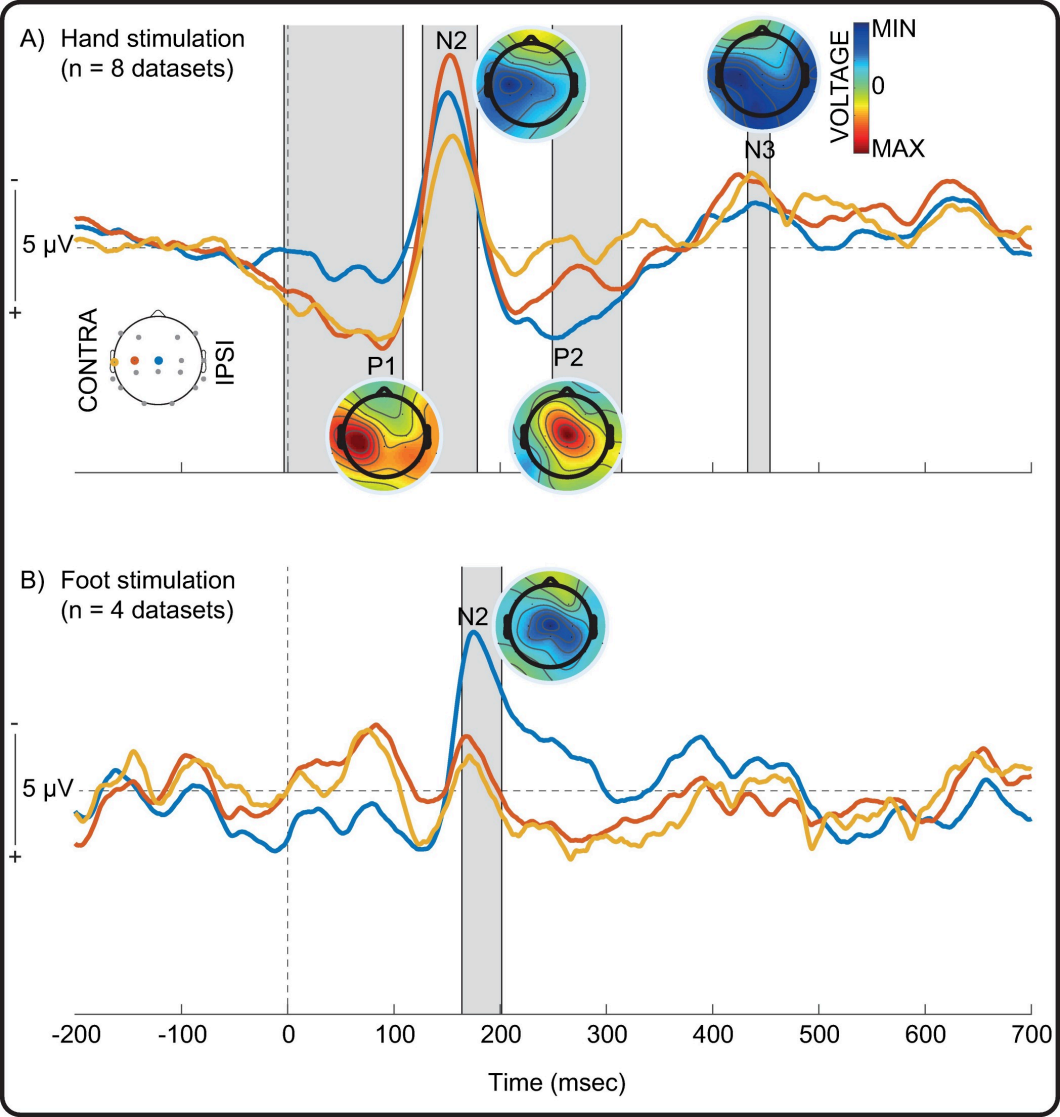
EEG response following tactile stimulation of hand and foot. Grand average of the EEG responses following tactile stimulation of A) hand, and B) foot recorded at the contralateral mid-temporal (yellow), contralateral central (red), and midline central (blue) channels. Grey shading represents significant deflections (p < .05) from baseline recorded at one or more of these sites. The time of the stimulus (0 ms) is marked by a dashed vertical line. Average scalp maps for the peak latencies of Negative (N) and Positive (P) peaks are also shown (symmetrically scaled).

Tapping the hand evoked a typical sequence of somatosensory evoked potentials: P1, N2, P2 and N3 (Fig 6). (N2 is labelled as such to distinguish it from a preceding N1 potential which can occasionally be appreciated but is typically ill-defined [12,34]). The latencies of the peaks were at approximately P90-N165-P255-N440 (significantly different from baseline -5-108 ms, 127–178 ms, 249–315 ms, and 433–454 ms). Tapping the foot also evoked the N2 potential (significantly different from baseline 164–201 ms) (Fig 6). The P1 and N2 potentials were maximal overlying the contralateral central region for the hand and midline central region for the foot, while the P2 potential evoked by hand stimulation was distributed at the midline central region, in line with previous reports [11,12,16,23,27,29,34–39]. The N3 potential evoked by hand stimulation was diffuse but maintained a contralateral emphasis.

#### Touching the face evokes a clear somatosensory response in the brain

**Fig 7:**
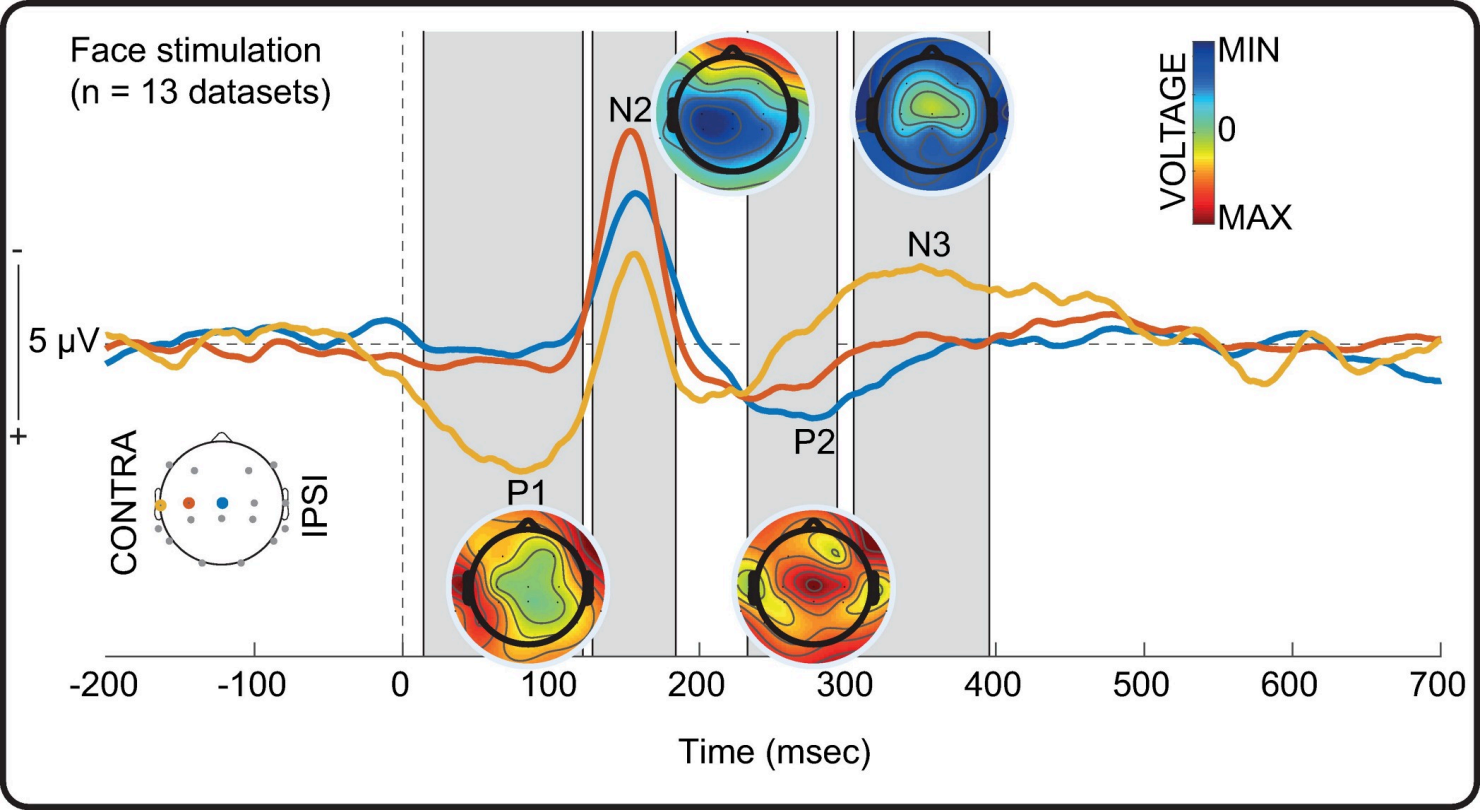
EEG response following tactile stimulation of the face. Grand average response recorded at the contralateral mid-temporal (yellow), contralateral central (red), and midline central (blue) channels. Grey shading represents significant deflections (p < .05) from baseline recorded at one or more of these sites. The time of the stimulus (0 ms) is marked by a dashed vertical line. Average scalp maps for the peak latencies of Negative (N) and Positive (P) peaks are also shown (symmetrically scaled).

Tapping the face evoked a clear somatosensory response in every infant (S2 Fig) comprising a similar sequence of somatosensory evoked potentials to hand stimulation except for the N3, which peaked slightly earlier at approximately N350 (significantly different from baseline 14–121 ms, 128–185 ms, 232–293 ms, and 304–396) (Fig 7). The P1 and N2 potentials were maximal overlying the contralateral temporal and contralateral central regions respectively, while the P2 potential was distributed at the midline central region as for hand stimulation. The topography of the N3 potential was diffuse.

## Discussion

Facial somatosensation is important for breast-feeding, but its development over the equivalent of human gestation is unknown. The importance of facial somatosensation in early life is indicated by its earlier development compared to caudal parts of the body in mammalian neonates which breastfeed [40–42]. Consistent with this, hand-to-mouth and facial movements are evident on ultrasound from just nine weeks of human gestation and are more prominent in pre-term infants than at full-term [43,44] (S2 Movie). These movements and their associated proprioceptive and tactile feedback may provide the necessary signalling for development of facial body maps in the cortex [45]. In order to investigate the maturation of facial somatosensory functions, we have developed an interface consisting of a novel wearable device that measures intensity and timing of gentle finger taps to skin in concurrence with EEG.

Our tests first demonstrated that the sensor is comfortable and intuitive to use. The force measurement and synchronisation with EEG was reliable and sensitive and allowed us to demonstrate the existence of a clear somatosensory response to tactile stimulation of the face in a group of late pre-term and early-term infants. This response comprised a typical sequence of somatosensory evoked potentials including the P1, N2, P2 and N3. Similar potentials have been evoked using electrical [11,12,23,34,46,47] or mechanical [16,36,48] stimulation of the limbs as also confirmed in this study.

Short-latency potentials (≤100 ms post-stimulus) including the P1 can be recorded from 29 weeks CGA [11,12,23,46] with contralateral central distribution following hand stimulation and midline central distribution following foot stimulation. They are thought to be generated from the limb’s corresponding somatotopic representation in primary somatosensory cortex, potentially Brodmann area 3b [49]. Consequently short-latency potentials represent the arrival of the peripheral afferent input and are therefore associated with intact somatosensory pathways and favourable neurodevelopmental outcome [38,50,51]. Here we show that the P1 evoked by mechanical stimulation of the face is more lateral than that evoked by hand stimulation, in line with the location of their respective cortical representations [52]. Identifying a topographical profile specific to facial stimulation is an important first step in studying somatosensory functioning which may underpin breastfeeding behaviours. Mechanical stimulation may be slightly less suited to capturing the earliest evoked and very low amplitude N1 potential (40 ms post-stimulus) owing to its relatively weak intensity and indistinct onset compared to electrical stimulation [36,48]. However, recent studies have shown that assessment of somatosensory evoked potentials which succeed the N1 can also evaluate somatosensory pathways and predict neurodevelopmental outcome in pre-term infants [29].

The N2 can be recorded from 31 weeks CGA and is the most prominent somatosensory evoked potential in pre-term infants, with contralateral central topography following hand stimulation and midline central topography following foot stimulation [33,53]. It is thought to be generated in the contralateral post-central gyrus until 38 weeks CGA, when involvement of the ipsilateral hemisphere emerges [33,54]. Here we support these findings and demonstrate that the N2 potential can also be consistently evoked by face stimulation from 33 weeks CGA with contralateral central distribution.

The P2 potential can be recorded from 35–38 weeks CGA with a symmetrical central midline distribution to stimulation of either hand or foot [11,16,33], and is likely generated by posterior parietal and secondary somatosensory cortex [29,33]. Here we show that this P2 potential can be evoked by facial stimulation in a cohort of just 35 weeks CGA (mean). The N3 potential is scarcely reported in the literature, although we have recently demonstrated that this potential emerges between 35–38 weeks CGA together with the P2 [33]. In this study we demonstrate that the topography of the N3 is diffuse in pre-term infants following either face or hand stimulation, and is not yet topographically organised at the central midline as in full-term infants [33].

Our sensor has the major advantage that it can be used to investigate facial somatosensory processing, indexed by somatosensory evoked potentials, while accurately controlling the site of stimulation and minimising the force exerted. In addition, the ability to wear the sensor on the finger allows us to measure somatosensory responses to naturalistic stimulation, such as a caregiver would provide. This is particularly important in light of recent evidence that positive tactile experience may benefit somatosensory development [55]. As with any human-delivered tactile stimulation, the specific force exerted varied across taps, which could be a potential limitation. However, the acceptability of this method with regard to infant comfort and parental expectations paves the way to track the development of facial somatosensory functioning across the equivalent of the third trimester of human gestation.

## Supporting information

S1 Methods Description of the first prototype of the touch sensor. (DOCX)

S2 Methods Values of the force applied for each stimulation in all subject. (XLS)

S1 Fig EEG response following tactile stimulation of the hand in a single infant. Grand average of the EEG responses recorded at the contralateral central channel. The time of the stimulus (0 ms) is marked by a dashed vertical line. Negative and positive deflections are denoted as N and P potentials respectively.
(TIF)

S2 Fig Individual EEG responses following tactile stimulation of the face. Individual (thin lines) and grand average (think line) responses recorded at the contralateral central channel for each of 13 stimulation trains from 7 infants, aligned according to N2 (yellow shading). The time of the stimulus (0 ms) is marked by a dashed vertical line.
(TIF)

S1 Movie Example of face stimulation which evokes a clear EEG response in an infant with corrected gestational age 37+1 weeks+days. EEG display-filtered: 0.2-20Hz. Sensitivity 25μV/mm.
(MP4)

S2 Movie Example of repetitive facial movements in an infant with corrected gestational age 30+6 weeks+days. Video plays at 1.00 followed by 0.23 real-time speed, for visualisation purposes.
(MP4)

S1 Results Dataset for subject104. (ZIP)

S2 Results Dataset for subject105. (ZIP)

S3 Results Dataset for subject106. (ZIP)

S4 Results Dataset for subject109. (ZIP)

S5 Results Dataset for subject110. (ZIP)

S6 Results Dataset for subject111. (ZIP)

S7 Results Dataset for subject112. (ZIP)
