# Supplementary material for: A novel sensor design for accurate measurement of facial somatosensation in pre-term infants
Source: PLoS One. 2018 Nov 16;13(11):e0207145. doi: 10.1371/journal.pone.0207145 (PMC6239299; doi:10.1371/journal.pone.0207145)
Supplement: S1 Methods — (DOCX) [file pone.0207145.s001.docx]

**S1_Methods**

The first prototype of the touch sensor included *i*) a force transduction compartment and *ii*) a sensor-recording system interface. Force transduction was obtained with a force sensor (TekScan FlexiForce A101) located at the fingertip of the index finger, converting the force applied by the user into variable resistance value $R_{s}$. Changes in $R_{s}$ are then converted into a voltage signal through a commercial amplifier (Phidgets Flexiforce Adapter) located on the dorsum of the hand, whose gain was tuned to detect forces up to 3000 mN. The voltage output of the amplifier was directly fed through an auxiliary line to the polygraphy system (Neuroscan^TM^ SynAmps 2) so that the force could be recorded concurrently with the EEG. A +5V power supply for the amplifier was obtained from the auxiliary lines of the SynAmps 2, therefore the $V_{\mathrm{OUT}}$ (0 to +5V) increase as more force is impressed on the sensor, up to the saturation value of the amplifier (i.e. the maximum force value detectable from the prototype).

The prototype was then tested on a single occasion with a pre-term infant (gestational age at birth 26+4 weeks+days, corrected gestational age at study 34+5 weeks+days) by clinical scientist KW. A series of 11 taps were delivered to the hand while wearing the touch sensor. Force deflections associated with each tap could be detected from the force recording. This allowed us to mark the occurrence of each tap on the concurrent EEG recording. We were then able to epoch the EEG according to the event timing and average responses within the subject leading to a clear somatosensory response associated to the tapping of the hand (S1_Fig).

Nevertheless, this first prototype suffered from the following limitations, which were addressed in the second design iteration presented in the main text:

1. The detectable force range did not allow to record very light taps.
2. The sensibility of the sensor did not allow to record very brief taps.
3. The load was not spread evenly across the sensor.
4. The use of wires to connect the sensor pins to the amplifier made the device insufficiently robust.
5. The sensor lay directly on the fingertip making the measurements unreproducible and inaccurate due to the skin deformation and the finger’s curvature.
6. The prototype was not calibrated so it was not possible to convert the force deflections associated with each tap to mN.
