# Supplementary figures and images for: A novel sensor design for accurate measurement of facial somatosensation in pre-term infants
Source: PLoS One. 2018 Nov 16;13(11):e0207145. doi: 10.1371/journal.pone.0207145 (PMC6239299; doi:10.1371/journal.pone.0207145)

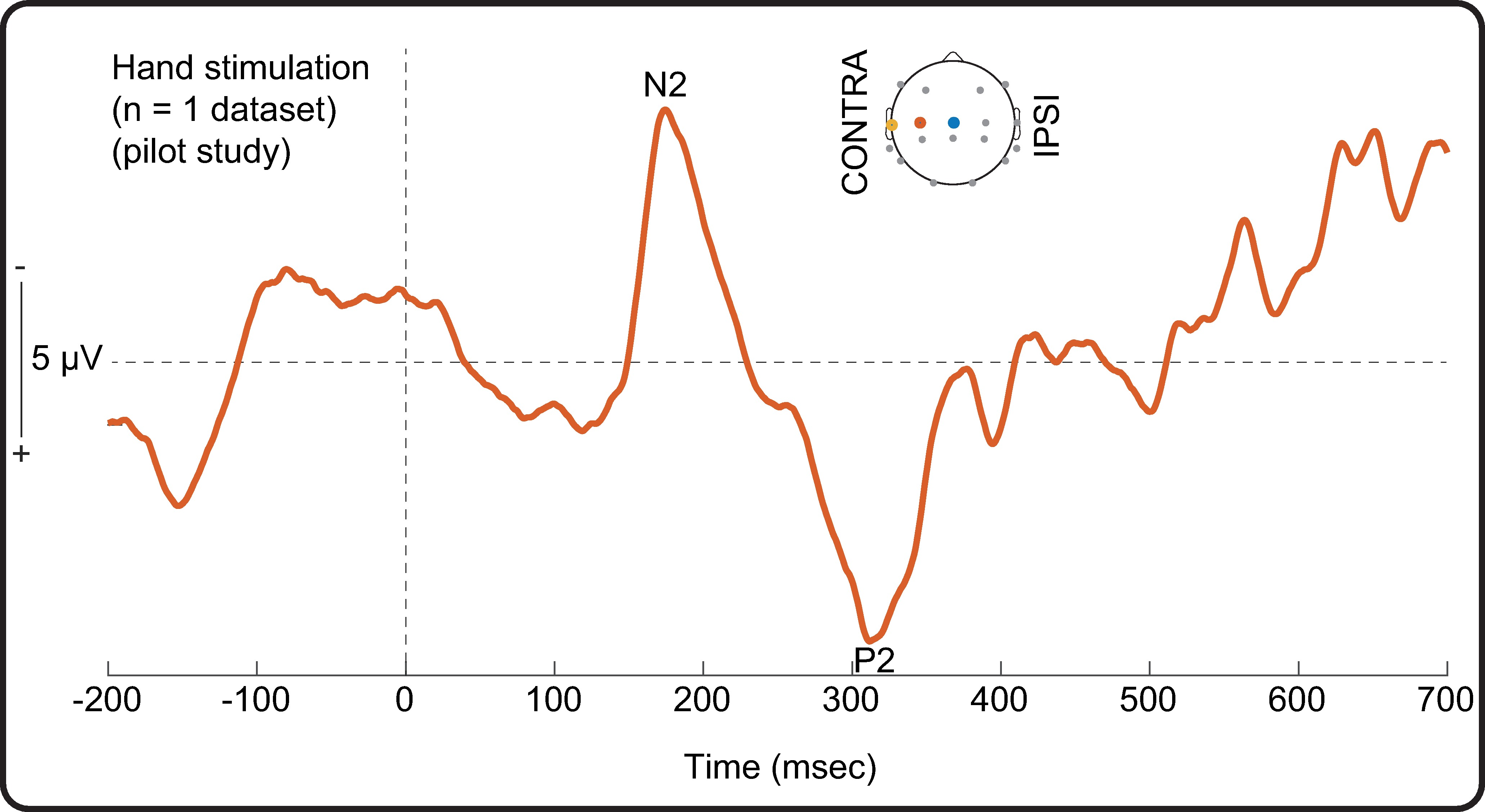

Supplement: S1 Fig — Grand average of the EEG responses recorded at the contralateral central channel. The time of the stimulus (0 ms) is marked by a dashed vertical line. Negative and positive deflections are denoted as N and P potentials respectively. (TIF) [file pone.0207145.s003.tif]

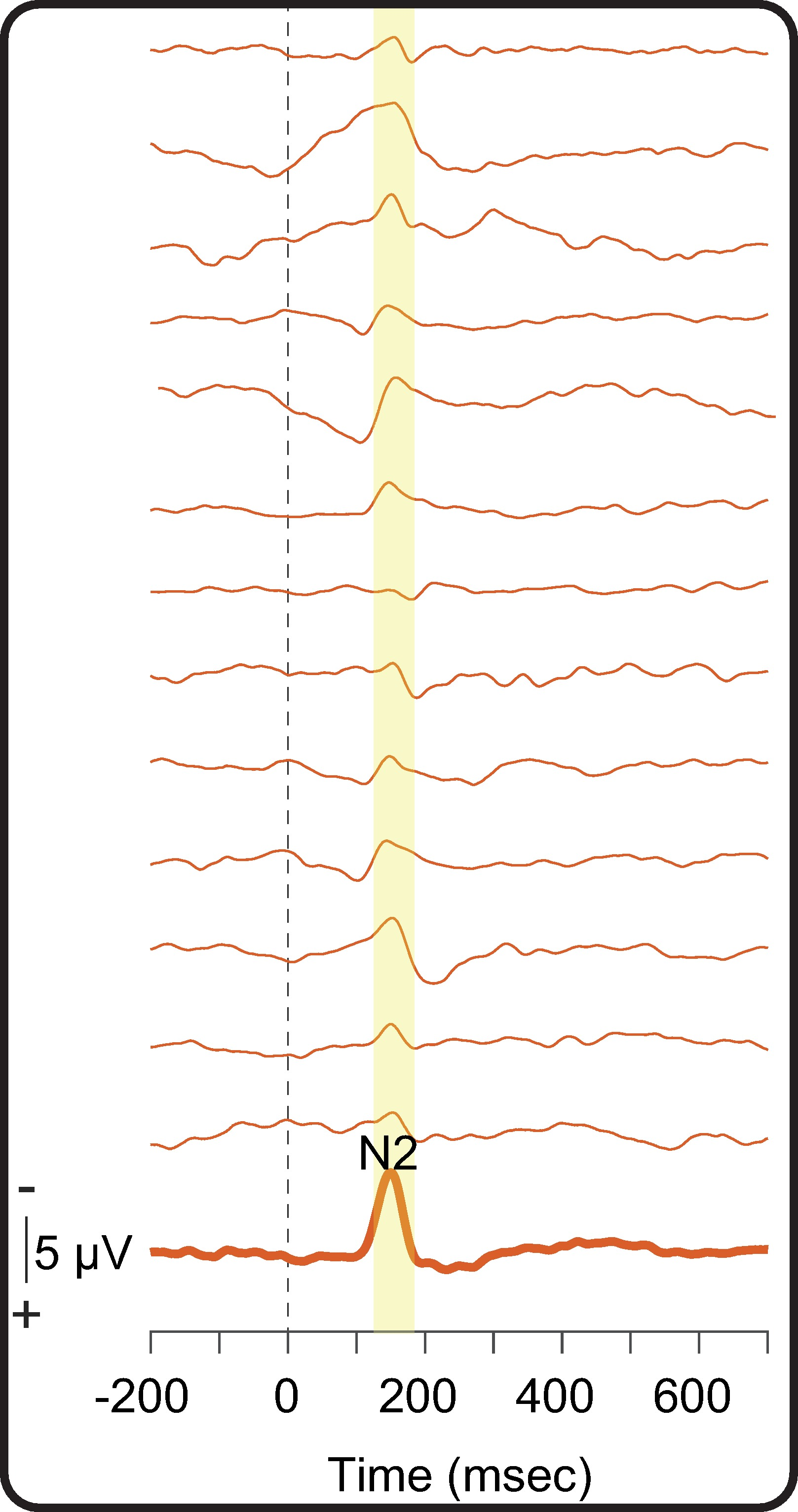

Supplement: S2 Fig — Individual (thin lines) and grand average (think line) responses recorded at the contralateral central channel for each of 13 stimulation trains from 7 infants, aligned according to N2 (yellow shading). The time of the stimulus (0 ms) is marked by a dashed vertical line. (TIF) [file pone.0207145.s004.tif]
